# Steroid Therapy and Steroid Response in Autoimmune Pancreatitis

**DOI:** 10.3390/ijms21010257

**Published:** 2019-12-30

**Authors:** Hiroyuki Matsubayashi, Hirotoshi Ishiwatari, Kenichiro Imai, Yoshihiro Kishida, Sayo Ito, Kinichi Hotta, Yohei Yabuuchi, Masao Yoshida, Naomi Kakushima, Kohei Takizawa, Noboru Kawata, Hiroyuki Ono

**Affiliations:** 1 Division of Endoscopy, Shizuoka Cancer Center 1007, Shimonagakubo, Nagaizumi, Suntogun, Shizuoka 411-8777, Japan; h.ishiwatari@scchr.jp (H.I.); ke.imai@scchr.jp (K.I.); y.kishida@scchr.jp (Y.K.); sa.ito@scchr.jp (S.I.); k.hotta@scchr.jp (K.H.); y.yabuuchi@scchr.jp (Y.Y.); ma.yoshida@scchr.jp (M.Y.); n.kakushima@scchr.jp (N.K.); k.takizawa@scchr.jp (K.T.); n.kawata@scchr.jp (N.K.); h.ono@scchr.jp (H.O.); 2 Genetic Medicine Promotion, Shizuoka Cancer Center 1007, Shimonagakubo, Nagaizumi, Suntogun, Shizuoka 411-8777, Japan

**Keywords:** autoimmune pancreatitis, IgG4, corticosteroid, treatment

## Abstract

Autoimmune pancreatitis (AIP), a unique subtype of pancreatitis, is often accompanied by systemic inflammatory disorders. AIP is classified into two distinct subtypes on the basis of the histological subtype: immunoglobulin G4 (IgG4)-related lymphoplasmacytic sclerosing pancreatitis (type 1) and idiopathic duct-centric pancreatitis (type 2). Type 1 AIP is often accompanied by systemic lesions, biliary strictures, hepatic inflammatory pseudotumors, interstitial pneumonia and nephritis, dacryoadenitis, and sialadenitis. Type 2 AIP is associated with inflammatory bowel diseases in approximately 30% of cases. Standard therapy for AIP is oral corticosteroid administration. Steroid treatment is generally indicated for symptomatic cases and is exceptionally applied for cases with diagnostic difficulty (diagnostic steroid trial) after a negative workup for malignancy. More than 90% of patients respond to steroid treatment within 1 month, and most within 2 weeks. The steroid response can be confirmed on clinical images (computed tomography, ultrasonography, endoscopic ultrasonography, magnetic resonance imaging, and ^18^F-fluorodeoxyglucose-positron emission tomography). Hence, the steroid response is included as an optional diagnostic item of AIP. Steroid treatment results in normalization of serological markers, including IgG4. Short- and long-term corticosteroid treatment may induce adverse events, including chronic glycometabolism, obesity, an immunocompromised status against infection, cataracts, glaucoma, osteoporosis, and myopathy. AIP is common in old age and is often associated with diabetes mellitus (33–78%). Thus, there is an argument for corticosteroid therapy in diabetes patients with no symptoms. With low-dose steroid treatment or treatment withdrawal, there is a high incidence of AIP recurrence (24–52%). Therefore, there is a need for long-term steroid maintenance therapy and/or steroid-sparing agents (immunomodulators and rituximab). Corticosteroids play a critical role in the diagnosis and treatment of AIP.

## 1. Introduction

Autoimmune pancreatitis (AIP), a unique subtype of pancreatitis, is characterized by focal or diffuse swelling of the pancreas and narrowing of the pancreatic duct, without marked upstream ductal dilation. AIP is particularly common among older males (mean age: 66.3 years), with a male/female ratio of 3.2:1 and an overall prevalence of 4.6 per 100,000 population, with a 1.4 per 100,000 annual incidence rate [1]. The symptoms of AIP vary, depending on which organs are associated. However, they generally include weak or mild abdominal pain, general fatigue, weight loss, and jaundice, with various extrapancreatic lesions [2,3,4,5]. AIP is often associated with diabetes (33–78%) due to the impact of chronic pancreatic inflammation on glucose metabolism [6,7,8,9,10,11].

AIP is categorized into two subtypes based on its histology: lymphoplasmacytic sclerosing pancreatitis (LPSP) (type 1 AIP) and idiopathic duct-centric pancreatic with granulocytic epithelial lesions (IDCP with GEL) (type 2 AIP) [3,12,13,14,15]. Table 1 provides information on distinctive clinical features of AIP, such as age, sex, geographic distribution, associated extrapancreatic lesions, and the steroid response and relapse rate. Type 1 AIP is the pancreatic manifestation of immunoglobulin G4 (IgG4)-related disease [16,17], and often develops in older males and is accompanied by systemic, inflammatory, sclerosing lesions, such as those seen in sclerosing cholangitis, interstitial pneumonia and nephritis, dacryoadenitis, and sialadenitis [4]. Type 2 AIP is more common among young or middle-aged patients, and is often associated with ulcerative colitis and rarely with Crohn’s disease [18,19,20,21]. Serum markers, including immunoglobulin G4 (IgG4), antinuclear antibody, and rheumatoid factor, are frequently elevated in type 1 AIP [12,13,22]. There are no reported serological makers for type 2 AIP.

AIP results in the formation of mass lesions in the pancreas. The differential diagnosis of AIP is vital to distinguish the disease from malignancies (i.e., pancreatic cancer (PC) and malignant lymphomas). A histological examination by endoscopic ultrasonography-guided fine needle aspiration biopsy (EUS-FNAB) [23,24,25] is mandatory before treatment commencement to avoid unnecessary surgery. The clinical diagnosis is based on the International Consensus Diagnostic Criteria [2,14], which consists of serological, imaging, and pathological findings, as well as the response to steroid therapy. The International Consensus Diagnostic Criteria are considered superior to other diagnostic criteria for AIP in terms of their sensitivity and specificity [26,27].

Oral corticosteroid therapy is commonly used for AIP, unless the cases have severe steroid intolerance [28,29]. Glycemic control may also be required in cases with diabetes beforehand or simultaneously with steroid treatment, and pancreaticobiliary drainage is done only in cases with obvious cholangitis or severe pancreatitis [30,31] (transpapillary biliary stenting should be limited in the necessary cases, as a prolonged period of stenting may promote pancreatic stone formation [30]). In cases of severe steroid intolerance or suspected intolerance, treatment with rituximab or immunomodulators is preferred. The treatment strategy can be tailored according to local conditions, as medical insurance for these drugs differs in each country [32].

## 2. Steroid Response in Diagnosis of AIP

The steroid response is listed as an optional diagnostic condition among the International Consensus Diagnostic Criteria for AIP [2], as such a response is hardly seen on clinical images obtained from PC cases [33]. Thus, the steroid response aids the diagnosis of atypical AIP cases, especially those with normal serum IgG4 [34]. However, as corticosteroid treatment is effective against IgG4-related inflammatory pseudotumors of the liver [35,36] and lung [37], it can thus further diagnose an AIP mimicking a PC with distant metastases [36]. In cases of AIP with modest pancreatic enlargement, the steroid response is sometimes unclear. In such cases, endoscopic retrograde cholangiopancreatography is needed to confirm subtle improvements in pancreaticobiliary ductal narrowing and to satisfy the diagnostic criteria [38].

Actual cases of mimickers of AIP and atypical AIP are listed in the following sections.

### 2.1. Mimickers of AIP

In contrast to the aforementioned cases, some neoplastic or malignant cases can mimic [39,40,41] or co-exist with AIP (IgG4-related diseases) [42]. PC is the most common malignancy that mimics focal-type AIP, although tumors of this type can usually be distinguished from AIP in image findings by poor enhancement while still in the delayed phase, as well as from marked dilation of the upstream pancreatic duct [2,3,43]. By contrast, pancreatic lymphoma looks like a diffuse-type AIP, as it reveals a sausage-like, enlarged pancreas with diffuse narrowing of the main pancreatic duct [39], together with elevated levels of serum soluble interleukin-2 (IL2) receptor [44]. However, in the daily clinics, we actually encounter malignancies that are difficult to differentiate from AIPs. For instance, a case of pancreatic head cancer, with upstream pancreatic swelling due to the obstructive pancreatitis, mimics diffuse-type AIP and can lure the physician into conducting a EUS-FNAB at the wrong site [45]. A case of a sausage-like pancreas with high-levels of serum IgG4 (344 mg/dL, normal: 5–105 mg/dL) and anti-DNA antibody (14 IU/mL, normal: <6.0 IU/mL) was identified as adenocarcinoma after evaluation of a forceps biopsy from the stenotic site of the main pancreatic duct [40]. We also need to bear in mind the possibility of a simultaneous co-existence of AIP (or IgG4-related pathology) and pancreatic malignancies; a small invasive cancer within the lesion of AIP [42], and a concomitant pancreatic mixed acinar-ductal adenocarcinoma; and follicular lymphoma accompanied with IgG4-related pathology [46]. In the international guideline, a persistent pancreatic mass is an indication of steroid therapy after the negative work up for pancreatic malignancies by EUS-FNA, even in an asymptomatic case [32].

The pathological mimickers of AIP or IgG4-related diseases, such as Castleman’s disease [47], follicular pancreatitis [48], anti-neutrophil cytoplasmic antibody-associated vasculitides [49,50], inflammatory myofibroblastic tumor [51], and lymphoma [52], are listed from the viewpoints of lymphoplasmacytic proliferation.

### 2.2. Atypical Cases of AIP and Their Steroid Responses

As mentioned, images of AIP typically show focal or diffuse pancreatic enlargement, together with a narrowing of the pancreatic duct in the corresponding area, but without marked dilation of the upstream pancreatic duct. Enhancement is poor in the enlarged pancreas at the arterial phase, but the attenuation is usually recovered at the delayed phase on dynamic CT [43]. A capsule-like rim is sometimes visible around the pancreas in cases of AIP, reflecting the marginal fibrosis [2,3,14]. An enlarged pancreas dramatically shrinks in response to corticosteroid therapy in ≥95% of AIPs [28,53,54]. Vascular invasion is widely believed to represent a malignant finding but is often seen in cases of AIP and is dramatically recanalized by steroid therapy [55,56].

A widespread consensus on AIP image interpretation has now reduced the number of cases with diagnostic difficulty or atypical AIPs; however, a variety of atypical AIPs have been reported. These include a 9 mm, well-demarcated pancreatic mass that was not affecting the main pancreatic duct [57]; a 2 cm mass protruding inside the main pancreatic duct and mimicking a main duct-type intraductal papillary mucinous neoplasm (IPMN) [58,59]; a 3 cm, low-echoic mass concomitant with a branch-type IPMN [60]; a multilocular cyst with progression of symmetric wall thickening during the time course mimicking cancerization of a branch-type IPMN [61]; and a pancreatic mass invading the ascending colon and mimicking invasive pancreatic carcinoma [62]. Many of these lesions were not accurately diagnosed, but some did not undergo unnecessary resection due to a negative workup for malignancies using EUS-FNAB and the sequential confirmation of a steroid response [60,62]. We should also bear in mind the possible occurrence of AIP or AIP-like pancreatitis due to treatment using an immune checkpoint inhibitor [63].

### 2.3. Development of Malignancies in the Course of AIP

Interval malignancies have also been reported in cases of AIP or IgG4-related diseases. For example, Shiokawa et al. [64] reported a recognition of cancer development in 14% of AIP cases over an average of 3.3 years of observation, with an obviously increased standardized incidence ratio (2.7) that was especially high in the first year of AIP diagnosis (6.1). Similarly, Ikeura et al. [65] reported three cases (4.8%) of PC development during an average of 95 months of follow up in 63 AIP patients, whereas Ishida et al. [66] reported the development of malignant lymphoma in the parotid gland seven months after the diagnosis of AIP. Even after successful discrimination of AIP from malignancies, clinicians must remain aware of the possible development of cancers [65,67,68] or lymphomas [68,69,70,71] during the follow up of the patients with AIP or IgG4-related diseases, as epidemiological [64,65,67,68,69,70] or genetic [71] risks have been reported.

## 3. Corticosteroid Treatments for AIP

### 3.1. Corticosteroid Therapy (Standard Steroid Therapy)

**Figure 1:**
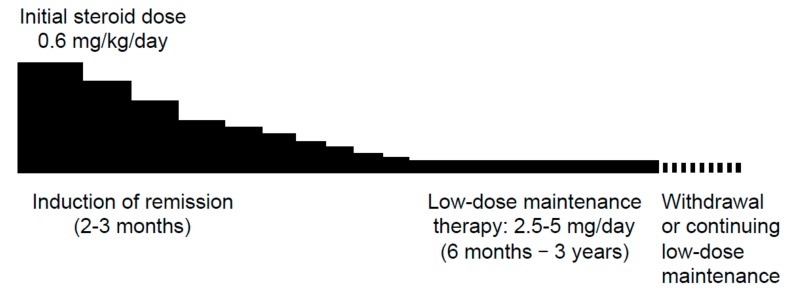
A timetable of standard oral corticosteroid therapy.

The major indication for steroid treatment is the presence of symptoms. The schedule for standard oral steroid treatment is as follows: an initial prednisolone dose of 0.4–0.6 mg/kg/day for 2–4 weeks, reduced by 5 mg/d every 1–2 weeks until 10–15 mg/day, and then gradually tapered to a maintenance dose of 2.5–5 mg/day over a period of 2–3 months (Figure 1) [28]. Maintenance treatment with low-dose steroids reduces but does not eliminate the risk of relapses. According to 2010 guidelines for type 1 AIP, after maintenance therapy for 6–12 months, further treatment tapering and withdrawal were recommended [28]. However, current guidelines recommend low-dose (5 mg/day) maintenance steroid treatment (MST) for 2–3 years to reduce the relapse rate (<30%) [29].

### 3.2. Rationale for Maintenance Steroid Therapy

The current guidelines are based mainly on the results of nationwide studies in Japan. Kubota et al. [29] retrospectively analyzed relapse rates in 510 patients (average age: 65.2 years; males: *n* = 393; females: *n* = 117 female) with type 1 AIP in a follow-up of an average of 61.1 months. In their study, oral intake of 2.5–5 mg/day of prednisolone for ≥6 months was defined as low-dose MST. The overall relapse rate in an MST 5 mg/day group (26%) was significantly lower than that in a non-MST group (45%, *p* = 0.023), and the relapse rate was even lower in a 2.5 mg/day MST group (43%, *p* = 0.001). In their study, the relapse rate almost reached a plateau after 7 years (43%) and remained unchanged after 10 years (47%). During the study period, possible steroid-related complications were recorded in 4% (20/510) of patients, with the majority of complications developing after 3 years. Several patients experienced poor glycemic control, which was treated with oral antidiabetic agents or insulin injections. Osteoporosis developed in 13 (2.5%) cases. Other complications were steroid myopathy (*n* = 1), fungal infections (*n* = 3), bacterial infections (*n* = 1), cerebral infarctions (*n* = 1), and atherosclerosis (*n* = 1). Many of these complications (50%) developed after the accumulated steroid amount exceeded 10,000 mg, and adverse events became severe (15/20) when MST was continued for > 5 years. In a Japanese nationwide randomized controlled trial, Masamune et al. [72] compared the relapse rate of patients receiving MST (5–7.5 mg/day) for ≥3 years with that of a cessation group (treatment discontinued after 26 weeks). The relapse rate was significantly higher in the cessation group (58%, 11/19 patients within 3 years) than that in the MST group (23%, 7/30 patients) (*p* = 0.011), despite no serious steroid-related events requiring steroid discontinuation in both groups. For long time, it had been believed that type 2 AIP rarely recurs after the initial steroid treatment [3,12,13,73]. However, the multicenter Dutch cohort study showed 27% (3/11) of recurrence in type 2 AIP during the median follow-up of 52 months, treatable by the restart of corticosteroid. They also described the necessity for MST as almost half of the AIP patients (55/107) during the median 74 months of follow-up [21].

MST recommendations are based mainly on the data in the aforementioned studies. However, most cases of AIP occur in elderly patients, and some corticosteroid-related events (e.g., cataracts, glaucoma, and osteoporosis) likely emerge after a long period. Further observations may be needed in these studies. To think of a good response by restarting corticosteroid therapy [28,29,32,74] and an increase in severe complications due to a high cumulative dose of steroids [75], relapse may not necessarily be weighted as a primary matter. As the risk factors predicting relapse have been intensively studied, limiting MST to high-risk patients may be a feasible treatment strategy.

Occasionally, an enlarged pancreas spontaneously shrinks without steroid treatment. Spontaneous regression is most likely to be seen in female type 1 AIP patients with biliary stent placement [76]. Hence, it may be better to follow the disease status for a few weeks in such cases. In principle, steroid treatment should be initiated after a diagnosis of AIP or at least after negative work up by EUS-FNAB, with facile steroid trials for diagnostic purposes avoided [2,14].

## 4. Steroid Response in Cases of AIP

### 4.1. Steroid Response Ratio and the Duration until Response Recognition

The majority of AIP patients respond to steroid treatment (steroid response), with reports of response rates of 97–100% [21,53,54]. This response can be detected in imaging studies as a shrinkage of the enlarged pancreatic parenchyma at 1 month after steroid initiation [54] and often within 2 weeks (86–100%) [33,54]. Therefore, an initial imaging study is recommended within 1–2 weeks after starting steroid therapy [77]. Response by rituximab can be obtained in cases of relapsed AIP within 15 days; however, it takes more by azathioprine as their treatment effect is weaker [78]. Usually, 3–6 months after steroid treatment initiation for AIP, the thickness of the enlarged pancreas has decreased to 60–70% of pretreatment phase [30].

### 4.2. Biomarkers for Assessing Steroid Response and Relapse

Serum IgG4 [13,16,22,79,80,81,82], IgE [83], soluble IL2-receptor [44] and peripheral blood eosinophils [83], memory B cells [84], and Th2 memory cells [85] have been reported as biomarkers of the disease activity of type 1 AIP and IgG4-related diseases. In particular, high level of baseline serum IgG4 [22,79,82,86] and IgE [83] are thought to be predictive markers of relapse. Increased Th2 memory cells may not directly reflect activity of IgG4-related diseases, but may result from the concomitant atopic manifestation [85].

### 4.3. Radiological and Ultrasonographic Evaluation for Assessing AIP Response to Steroids

**Figure 2:**
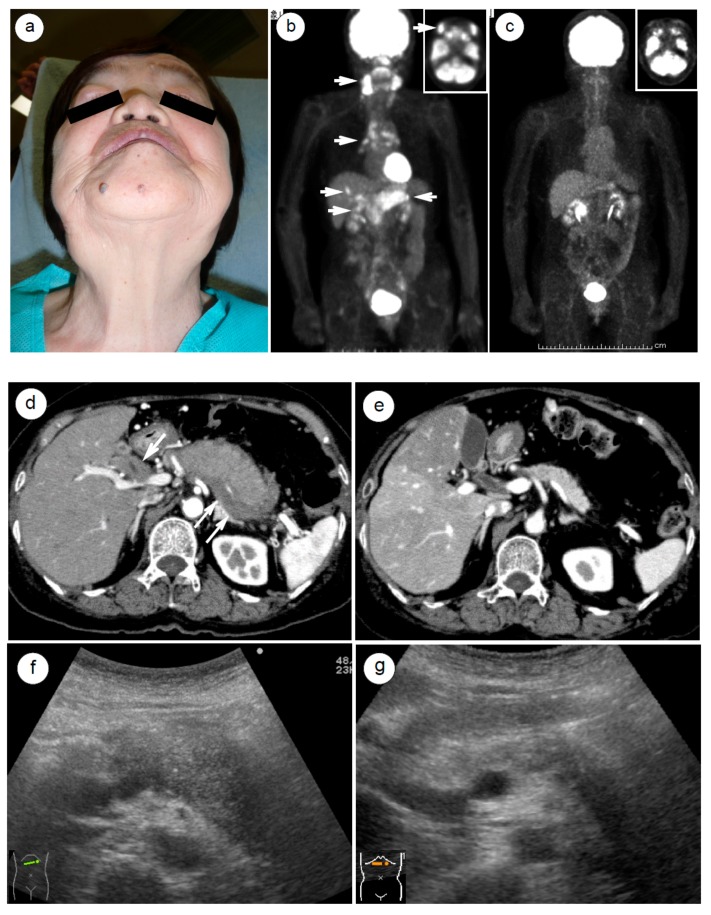

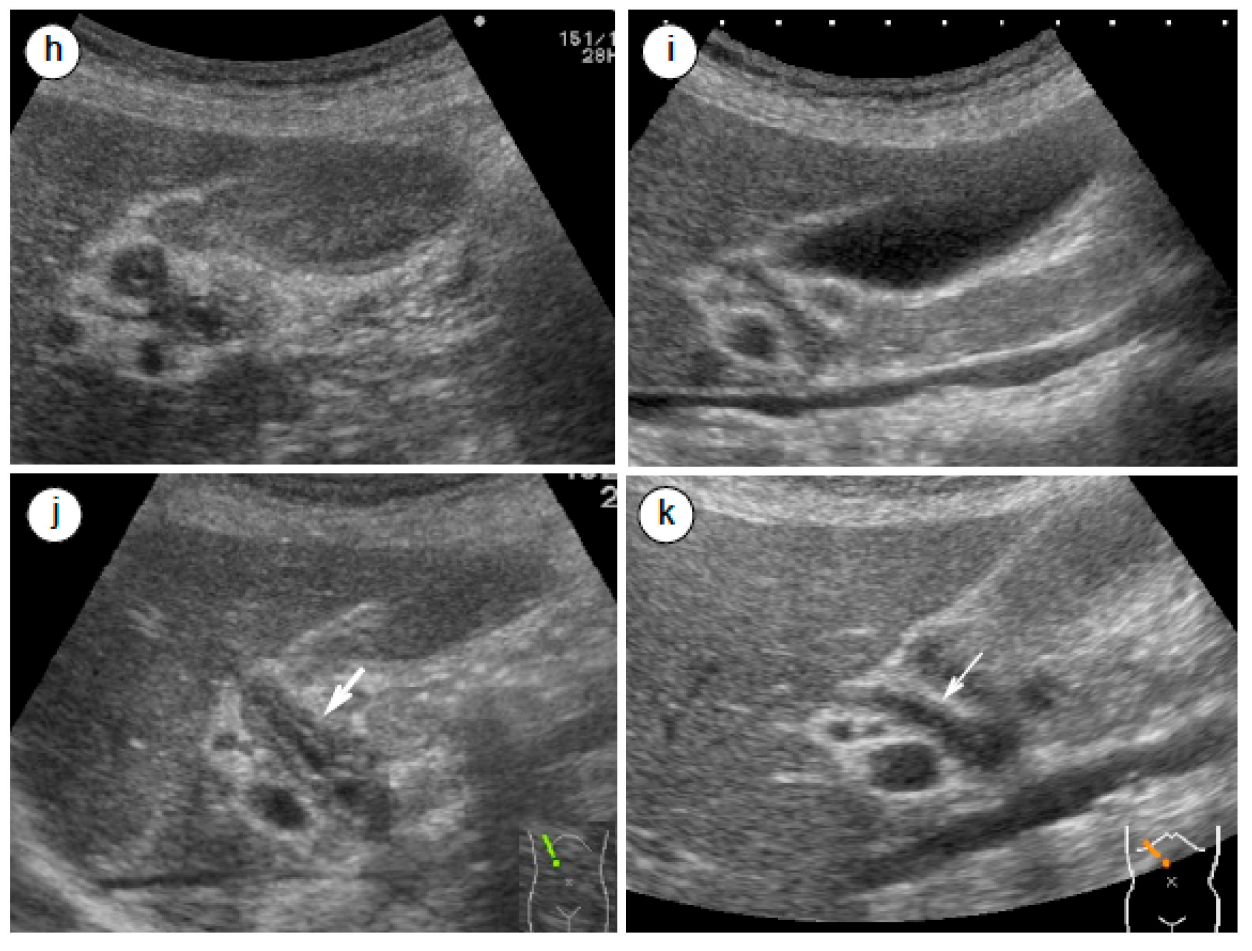
A case of Mikulicz disease with autoimmune pancreatitis (AIP) accompanied by multiple immunoglobulin G4 (IgG4)-related systemic lesions in a 70 year-old female patient. (**a**) Visualization by 18F-fluorodeoxyglucose-positron emission tomography (FDG-PET) of systemic inflammatory lesions with abnormal FDG uptake (the arrow heads indicate from the top to the bottom: dacryoadenitis, sialadenitis, mediastinum lymphadenopathy, hepatic pseudotumor, autoimmune pancreatitis, and choledocho-chlecystitis. (**b**) Disappearance of the lesions 3 months after steroid initiation. (**c**) Computed tomography before steroid treatment demonstrated an enlarged pancreas with a capsule-like rim (thin arrow) and markedly thickened hilar bile duct (thick arrow). (**d**) Computed tomography 1 year after steroid initiation revealed pancreatic parenchymal shrinkage and improved bile duct thickness. (**e**) Abdominal ultrasonography before (**f**,**h**,**j**) and 2 months after (**g**,**i**,**k**) steroid therapy showed a dramatic improvement in the size of the pancreas, as well as wall thicknesses of the gallbladder up to the hilar bile duct (white arrows).

**Figure 3:**
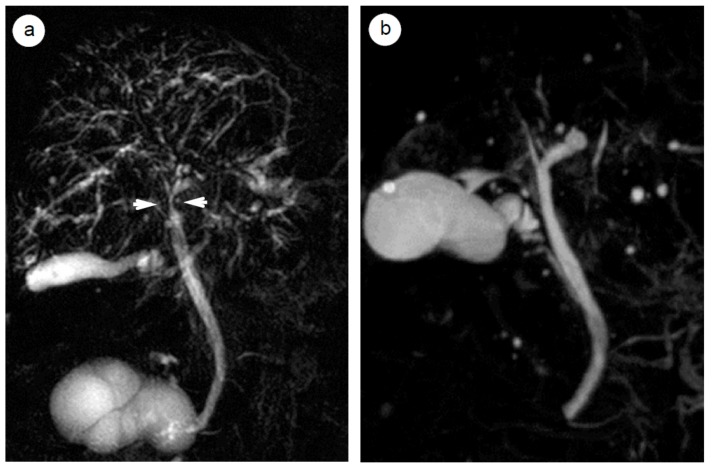
A case of focal-type AIP with sclerosing cholangitis at the hilar and intrahepatic bile ducts in a male 70 year-old patient. Magnetic resonance cholangiopancreatography before steroid treatment showed (**a**) multiple biliary stenosis at the hepatic hilum (arrow heads) and intrahepatic bile ducts, with improvements 3 months after steroid therapy (**b**).

The steroid response can be detected by imaging studies [54,55,87,88]. In terms of imaging studies, 18F-fluorodeoxyglucose-positron emission tomography (FDG-PET) [88] can visualize the steroid response of systemic lesions associated with AIP in a single whole body view, as shown in Figure 2a–c, which depict a case of Mikulicz disease [88]. Figure 2d,e shows early-phase contrast computed tomography (CT) imaging results, which depict dramatic shrinkage of the enlarged pancreatic parenchyma and disappearance of a capsule-like rim and extrapancreatic lesions [55]. Figure 2f–k show abdominal ultrasound (US) images of IgG4-associated lesions in the abdomen [54]. Figure 3a,b shows magnetic resonance cholangiopancreatography (MRCP) images of a normalized pancreaticobiliary duct, which was locally and diffusely narrowed by an enlarged pancreas and thickened biliary tract [87].

As described above, a steroid response can be recognized in various image modalities and is listed as an optional diagnostic condition; therefore, it is a critical diagnostic test for cases in which a definitive diagnosis of AIP cannot be reached. Enhanced CT is a standard modality for high resolution visualization of all AIP-associated lesions in thin-sliced, cross-sectional images without a blind area. Multidetector-row CT can construct maximum intensity projections and multi-planar reconstruction images that project rotatable sagittal and coronal views that provide a three-dimensional depiction of human anatomy. A differential diagnosis from other pancreatic diseases requires high-resolution views with detailed information of the contrast attenuation [89]. For this reason, enhanced CT is the most widely used modality in many clinical departments.

FDG-PET is a suitable modality for identifying systemic lesions associated with AIP in a whole body view. We previously demonstrated that abnormal uptakes of FDG by IgG4-related lesions were minimized or eliminated after steroid therapy [88]. PCs and malignant lymphomas will also sometimes display abnormal FDG uptakes; therefore, this modality can be effective for discriminating AIP from its neoplastic mimickers. For example, Shigekawa et al. [90] demonstrated that about 90% of PCs and AIPs show abnormal FDG uptakes at the pancreas (SUV max: 6.8 in AIPs and 7.7 in PCs), but a 1 week steroid therapy could weaken or eliminate FDG uptake in all the AIPs, whereas uptake was unchanged in PCs. Hence, sequential FDG-PET images can be used to evaluate the inflammation level of AIP, as well as to differentiate malignancies from AIPs. The drawbacks are the high cost and the high level of radiation exposure.

US is a handy imaging tool for visualizing the emergence and disappearance of most types of IgG4-related inflammatory lesions in the abdomen and body surface [54]. The advantages of US are its versatility, low cost, portability, and convenience, as well as its nonradiative nature that allows even pregnant women to undergo examination. US is utilized in practice for many IgG4-related lesions, including those in the salivary glands, thyroid, breast, urinary tract, aorta, pancreatobiliary tract, other abdominal solid organs, and superficial lymph nodes [54]. However, ultrasound is hindered by the gases that occupy the gastrointestinal tract and lung, and imaging is poor in obese individuals. The quality of US observation depends largely on the target organs, the patient’s anatomy, the time available for the examination, and the skill of ultrasound technician.

Endoscopic ultrasonography (EUS) can be used to visualize the steroid response in the pancreatic parenchymal, as well as the ductal findings (e.g., parenchymal hypertrophy, hyperechoic foci, hyperechoic strand, lobularity, and high-echoic margin of the main pancreatic duct) that are often seen in early chronic pancreatitis and that often respond to steroid therapy [91]. These precise findings cannot be clearly seen with abdominal US, suggesting this as a possible advantage of EUS. Accurate sonographic information that can discriminate other pancreatic lesions can also be obtained using contrast enhancement [92] and elastography [93]. The drawbacks of this modality are the necessity for conscious sedation, it is an operator-dependent examination quality, and blind areas can exist after some upper gastrointestinal tract reconstruction surgeries.

MRCP [87] can be used to visualize pancreatobiliary tracts without radiation exposure and without severe complications such as pancreatitis that can possibly occur in response to endoscopic retrograde cholangiography. Recognition of a subtle difference in the width of the main pancreatic duct, either a narrowing or an obstruction of the duct, is critical for the differential diagnosis between AIP and pancreatic cancer, and MRCP, unlike ERCP, cannot definitively elucidate this difference. However, MRCP is useful for confirmation of the steroid response, as a previously non-visualized narrowed pancreatic duct becomes visible after steroid treatment [87]. Contrast-enhanced MRI is also helpful for differentiation between AIPs and PCs, as the contrast of the lesion (between the masses and surrounding normal pancreata) was significantly different between AIPs and PCs, especially at the arterial phase [94]. Preserved vascular attenuation [94], homogeneous enhancement pattern, duct penetrating sign, and apparent diffusion coefficient (ADC) value [95] have been reported to be key features of AIP.

### 4.4. Steroid Response in Pancreatic Cystic Lesions

**Figure 4:**
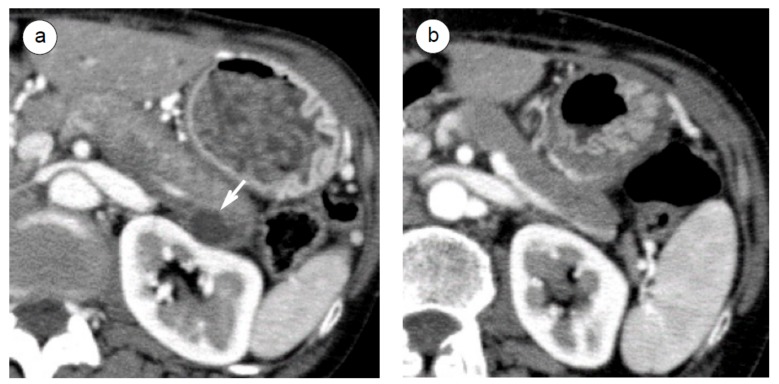
A steroid response in a pancreatic cyst of an AIP patient. Computed tomography demonstrated a unilocular cyst (arrow) at the pancreas tail, 15 mm in size, at the initial diagnosis of AIP (**a**). The cyst disappeared after steroid initiation (**b**).

Pancreatic cystic lesions are sometimes associated with AIP (10–22%) [38,96,97,98], with most of these lesions being retention cysts [99] or pseudocysts [98]. These cystic lesions respond well to steroids and are minimized or disappeared in 67–78% of cases [38,96,97,98] (Figure 4a,b). Although most small-sized cysts respond well to steroids, larger cysts (>55 mm) tend to be incurable without endoscopic treatment [96,98]. However, mucinous neoplastic cysts and cancer-associated cysts that often appear in multilocular cysts are resistant to steroid treatment, in contrast to steroid-responsive unilocular cysts in cases of AIP-associated pseudocysts, and thus careful examinations or observations are needed for them [38]. In cases of clinical emergencies, endoscopic drainage, such as transpapillary naso-pancreatic duct drainage [31] or transluminal pancreatic cyst drainage [96,100,101], are recommended when associated with severe abdominal pain [31] and/or infection [100]. The recommended treatment for hemorrhagic cysts is usually surgery [102] or a transvascular intervention.

### 4.5. Extrapancreatic Lesions

A group of extrapancreatic lesions, including dacryoadenitis, sialoadenitis [103], hilar lymphadenopathy [88], interstitial pneumonitis [104], sclerosing cholangitis [21,35,54], retroperitoneal fibrosis [105], and tubulointerstitial nephritis [106], are thought to have a close association with type 1 AIP, whereas inflammatory bowel diseases (e.g., ulcerative colitis and Crohn’s disease) are associated with type 2 AIP [2,4,12,14,21]. Another group categorized as possible AIP-associated lesions and other possible IgG4-related lesions are listed in Table 2. The latter lesions demonstrate the aggregation of IgG4-positive cells, but the full spectrum of IgG4-related pathology is not always confirmed (e.g., obliterative phlebitis and storiform fibrosis are sometimes lacking) [17].

### 4.6. Clinical Emergency in Cases of AIP or IgG4-Related Diseases

Some AIP-associated extrapancreatic lesions cause severe symptoms or represent a clinical emergency if left untreated. These include facial configuration changes due to dacryoadenitis (Mikulicz disease, Figure 2a) [103], vomiting and loss of consciousness due to hypophysitis [107], dyspnea due to pericarditis-induced effusion and heart failure [130,131,132], dysuria due to a urinary tract obstruction by retroperitoneal fibrosis [105], rupture of an inflammatory aortic aneurysm and periaortitis [35,54,132], and bleeding of gastric varices due to splenic vein obstruction by pancreatic mass involvement [55,133]. Clinicians must bear in mind that these aggressive conditions may occur in cases of type 1 AIP or IgG4-related diseases and that corticosteroids are effective and easy to use, especially in the early stage or prior to an emergency.

By contrast, some of our cases of IgG4-related diseases demonstrated adverse events after steroid treatment, namely, a case of IgG4-related mesenteric pseudotumor causing intestinal perforation due to the excessive steroid response [115] and a case of IgG4-related hepatic pseudotumor developing an abscess, probably due to the combined hyperglycemic and ischemic conditions [120]. Clinicians also need to stay aware of any adverse events that might possibly occur after steroid therapy.

## 5. Steroid Therapy for Diabetic Control in AIP Patients

Various adverse events associated with long-term low- to medium-dose oral corticosteroids have been reported: osteoporosis, myopathy, glucose intolerance and diabetes, fat redistribution, suppression of sex hormone secretion, dyslipidemia, atherosclerosis, cardiovascular diseases, hypertension, alopecia, cutaneous atrophy, acne, cataract, glaucoma, peptic ulcer, infections, psychosis, mood disturbances, headaches, vertigo, and tinnitus [134]. Many studies in the last decade have investigated the effect of corticosteroid treatment among AIP patients on diabetes [7,8,9,10,11,135]. According to a number of studies, many AIP patients also have diabetes mellitus (DM) (33–78%) at the time of their initial AIP diagnosis [6,7,8,9,10,11]. A nationwide survey in Japan in 2006 found that 55% of patients with AIP had simultaneous onset of DM [9]. Furthermore, 36% of AIP patients who had DM prior to AIP onset improved glycemic control by standard corticosteroid therapy [9]. These findings were supported by 3 year follow-up data, which revealed a diabetic improvement after steroid treatment in 63% (10/16) of patients [10]. Hirano et al. [7] applied the glucagon tolerance test (ΔCPR), insulin secretion test (HOMA-beta), and insulin resistance test (HOMA-R) in 47 patients with AIP. They assessed glucose tolerance via the ΔCPR test 1 month after steroid administration, and HOMA-beta and HOMA-R were examined during the following 60 months. Glucose tolerance improved in 13% of patients and was aggravated and unchanged in 19% and 68% of patients, respectively. Insulin secretion (HOMA-beta) improved significantly in 44–56% of patients, whereas insulin resistance was significantly aggravated (HOMA-R: 1.30–1.78). On the basis of these data and that of another study [11], the authors recommended steroid treatment at an early stage, especially for AIP patients with glucose intolerance. Masuda et al. [8] examined the diabetic status of 31 AIP patients who underwent steroid treatment and concluded that new onset of diabetes or worsened control of diabetes was common in patients with an atrophic pancreas after steroid induction. To sum up these study results, steroid treatment is recommended for AIP patients with diabetes. However, further studies are needed to clarify the pretreatment characteristics of patients who will not benefit from steroid treatment.

## 6. Recurrence of AIP and Factors Associated with Recurrence

As mentioned above, relapse occurs in 24–52% of type 1 AIP cases [22,29,72,73,74,79,80,81,136,137,138,139,140,141,142,143,144] and 0–27% of type 2 AIP cases [21,73,78] after cessation of corticosteroid treatment or dose tapering of corticosteroids. Identification of characteristics or risk factors for relapse is critical, as unnecessary steroid treatment in patients in whom recurrence is unlikely should be avoided. To date, several factors for predicting AIP relapse have been reported. These include a high level of serum IgG4 [22,79]; extrapancreatic lesions [78,80,136,137], especially proximal bile duct stenosis [73,136,138]; retroperitoneal fibrosis [139]; dacryoadenitis/sialadenitis [140]; jaundice [141]; discontinuation of steroid therapy [138,142,143]; a small reduction in the level of serum sIgG4 3–4 months after steroid initiation [80,81]; and a low level of pancreatic shrinkage following steroid treatment [144]. Maintenance corticosteroid therapy, with or without steroid-sparing agents, is recommended for type 1 AIP patients with factors predictive of relapse, although the therapeutic period is unclear.

## 7. Treatment Strategies for Steroid Refractory Cases

A small proportion of AIP patients exhibit no steroid response [53,54,74]. In other AIP patients, long-term steroid treatment is contraindicated or patients are refractory to standard corticosteroid therapy. In such cases, rituximab is a good alternative therapeutic choice [78,145,146]. A recent French study recommended to use rituximab for the treatment of recurrent AIP cases, as an efficacy rate was significantly higher by rituximab (94%, 16/17) than immunomodulators (azathioprine etc.) (67%, 14/21; *p* = 0.03) [78]. However, immunomodulators (azathioprine, cyclosporine A, and rapamycin) [21,147] are relatively cheaper and can be used to reduce the lifetime cumulative steroid dosage [32,145]. Unlike rituximab, monotherapy with immunomodulators is not fully effective [145]. Thus, these steroid-sparing agents are used in combination with low-dose steroids [145]. Disease-modifying antirheumatic drugs (DMARDs), such as methotrexate [148] and tacrolimus [149], are also thought of as a treatment choice for the steroid-resistant cases.

The use of immunosuppressants, such as immunomodulators, rituximab, and corticosteroids, in patients with occult hepatitis B virus infection (serum anti-Hepatitis B surface (HBs) antibody positive or anti-Hepatitis B core (HBc) antibody positive and HBs antigen negative) should be carefully monitored by quantification of serum hepatitis B virus-DNA, as the virus can be reactivated by immunosuppressive agents [150].

Although rituximab and immunomodulators are used in Western countries, they are not commonly used in Japan because they are not covered by medical insurance plans. In patients refractory to standard steroid therapy in Japan, steroid mini-pulse therapy (two courses of methylprednisolone 500 mg/day for 3 day, with a 4 day interval) is used [151,152].

## 8. Conclusions

Corticosteroids are the standard treatment for symptomatic cases of AIP. Steroid therapy also needs to be considered for asymptomatic patients with diabetes, as it improves glycemic control in many patients in the long term. Although a simple steroid trial before the negative work up of malignancies should be prohibited, a steroid response will support the diagnosis of AIP and is incorporated into the optional diagnostic criteria of AIP. Prolonged low-dose maintenance therapy (5 mg/day for 3 years) is recommended for AIP, especially for patients with known risks for recurrence. However, the risk of corticosteroid-associated adverse events, as well as the lifetime cumulative steroid dose, must be considered. To reduce the risk of adverse events and the lifetime cumulative steroid dose, the use of steroid-sparing agents (immunomodulators and rituximab) is an alternative treatment strategy.

## Figures and Tables

**Table 1:** Characteristics of type 1 and type 2 AIP.

	Type 1 AIP	Type 2 AIP
Distribution	Asia > USA, Europe	Europe > USA > Asia
Age at onset	60s–70s	40s-50s
Sex	Male >> Female	Male = Female
Symptoms	Jaundice, Abdominal pain	Jaundice, Abdominal pain
Serology	IgG4, IgG, Autoantibodies	(−)
Pancreatic images	Enlarged (focal, diffuse)	Enlarged (focal, diffuse)
Pancreatic histology	LPSP *	IDCP with GEL ^#^
Extrapancreatic lesions	Sclerosing cholangitis, Sialoadenitis, Retroperitoneal fibrosis, Interstitional nephritis, etc.	Inflammatory bowel disease
Steroid response	Mostly respond	Mostly respond
Relapse rate	24–52%	0–27%

AIP: autoimmune pancreatitis, IgG: immunogloblin G, * LPSP: lymphoplasmacytic sclerosing pancreatitis, ^#^ IDCP with GEL: idiopathic duct-centritic pancreatitis with granulocyte epithelial lesion.

**Table 2:** Extrapancreatic lesions associated and possibly associated with AIP or IgG4-related lesion.

Close Association	Possible Association with Type 1 AIP	Possible Association with IgG4-Related Lesion
with type 1 AIP	hypophysitis [107]	skull and vertebral lesions [108,109,110]
dacryoadenitis [103]	neurosensory hearing loss [4]	orbital lesions [109]
sialoadenitis [103]	chronic thyroiditis [4]	meningitis [111]
hilar lymphadenopathy [88]	inflammatory pseudotumors	esophagitis [112]
interstitial pneumonitis [104]	breast [113]	sclerosing mesenteritis [114,115]
sclerosing cholangitis [21,35,54]	lung [116,117]	Rosai-Dorfman disease [118,119]
retroperitoneal fibrosis [105]	liver [35,36,104,120]	gastroenteritis [121]
tubulointerstitial nephritis [106]	gastric ulcer [4]	vasculitis [122]
	swelling of the papilla of Vater [123]	neuropathy [124]
with type 2 AIP	hepatopathy [35]	myopathy [125]
inflammatory bowel diseases [2,4,12,14] (ulcerative colitis and Crohn’s disease)	aortitis [35]	dermatitis [126]
	prostatitis [4,88]	chondritis [127]
	Schonlein-Henoch purpura [4,128,129]	
	autoimmune thrombocytopenia [4,128,129]	

AIP: autoimmune pancreatitis.
